# Food insecurity and violence in a prospective cohort of women at risk for or living with HIV in the U.S.

**DOI:** 10.1371/journal.pone.0213365

**Published:** 2019-03-06

**Authors:** Amy A. Conroy, Mardge H. Cohen, Edward A. Frongillo, Alexander C. Tsai, Tracey E. Wilson, Eryka L. Wentz, Adaora A. Adimora, Daniel Merenstein, Ighovwerha Ofotokun, Lisa Metsch, Mirjam-Colette Kempf, Adebola Adedimeji, Janet M. Turan, Phyllis C. Tien, Sheri D. Weiser

**Affiliations:** 1 Center for AIDS Prevention Studies, University of California, San Francisco (UCSF), CA, United States of America; 2 Department of Medicine, Stroger Hospital, Chicago, IL, United States of America; 3 Department of Health Promotion, Education, and Behavior, University of South Carolina, Columbia, South Carolina, United States of America; 4 Chester M. Pierce, MD Division of Global Psychiatry, Massachusetts General Hospital, Boston, MA, United States of America; 5 Department of Community Health Sciences, State University of New York Downstate Medical Center, School of Public Health, Brooklyn, NY, United States of America; 6 Bloomberg School of Public Health, Department of Epidemiology, Johns Hopkins University, Baltimore, MD, United States of America; 7 School of Medicine and UNC Gillings School of Global Public Health, University of North Carolina at Chapel Hill, Chapel Hill, NC, United States of America; 8 Department of Family Medicine, Georgetown University Medical Center, Washington, DC, United States of America; 9 School of Medicine, Emory University, Atlanta, GA, and Grady Healthcare System, Atlanta, GA, United States of America; 10 Department of Sociomedical Sciences, Mailman School of Public Health, Columbia University, New York, NY, United States of America; 11 School of Nursing, School of Public Health and School of Medicine, University of Alabama at Birmingham, Birmingham, AL, United States of America; 12 Dept. of Epidemiology and Population Health, Albert Einstein College of Medicine, Bronx, NY, United States of America; 13 Department of Health Care Organization and Policy, School of Public Health, University of Alabama at Birmingham, Birmingham, AL, United States of America; 14 Department of Medicine, UCSF and Medical Service, Department of Veteran Affairs Medical Center, San Francisco, CA, United States of America; University of Texas Medical Branch at Galveston, UNITED STATES

## Abstract

**Background:**

Food insecurity and violence are two major public health issues facing U.S. women. The link between food insecurity and violence has received little attention, particularly regarding the temporal ordering of events. The present study used data from the Women’s Interagency Human Immunodeficiency Virus Study to investigate the longitudinal association of food insecurity and violence in a cohort of women at risk for or living with HIV.

**Methods:**

Study participants completed six assessments from 2013–16 on food insecurity (operationalized as marginal, low, and very low food security) and violence (sexual or physical, and psychological). We used multi-level logistic regression, controlling for visits (level 1) nested within individuals (level 2), to estimate the association of experiencing violence.

**Results:**

Among 2,343 women (8,528 visits), we found that victims of sexual or physical violence (odds ratio = 3.10; 95% confidence interval: 1.88, 5.19) and psychological violence (odds ratio = 3.00; 95% confidence interval: 1.67, 5.50) were more likely to report very low food security. The odds of experiencing violence were higher for women with very low food security at both the current and previous visit as compared to only the current visit. HIV status did not modify these associations.

**Conclusions:**

Food insecurity was strongly associated with violence, and women exposed to persistent food insecurity were even more likely to experience violence. Food programs and policy must consider persistent exposure to food insecurity, and interpersonal harms faced by food insecure women, such as violence.

## Introduction

Food insecurity, defined as having limited access to food and ability to acquire food [1], and physical, sexual, and psychological violence are two major public health problems affecting women in the United States (U.S.). Nationally-representative data show that female-headed households experience significantly higher rates of food insecurity as compared to households in general (30% versus 13%) [2]. Women in the U.S. also experience high rates of violence with at least 25% of women having experienced physical or sexual violence over their lifetime[3]. Food insecurity can have significant impacts on women’s mental and physical health including depression and substance abuse, human immunodeficiency virus (HIV) infection, and HIV-related morbidity and mortality through nutritional, mental health (e.g., depression), and behavioral (e.g., non-adherence to HIV medication) pathways [4]. Similarly, violence can lead to physical injury, chronic disease, depression, post-traumatic stress disorder, substance abuse, and sexually-transmitted infections [5, 6]. Rates of violence and food insecurity among HIV-positive women are high. Between 14–20% of U.S. women reported physical violence in the past six months [7] and an estimated 50% of HIV-positive individuals, including women, are food insecure [4].

Decades of research has demonstrated a consistent link between markers of poverty such as income, employment, and education, and violence against women [8, 9]. Poverty may constrain women’s power in their relationships due to economic dependence on partners, making women less able to leave an abusive relationship. It is also plausible that financial difficulties serve as a trigger for violence in families or couples through the path of stress [9, 10]. Less research has examined this association specifically for food insecurity—which is related to, but conceptually different from, poverty. Economic abuses in the household have been linked to women’s food insecurity and these same conditions may precipitate violence [11].

There is evidence that food insecurity and violence are positively associated. According to the Centers for Disease Control, women who reported being food insecure in the past 12 months experienced higher levels of rape and physical violence than women who reported being food secure [3]. Of the small number of studies on the association between food insecurity and violence, most consist of cross-sectional studies with people receiving public assistance [12, 13], small community-based samples [14], or people outside of the US [11, 15, 16]. In a few cross-sectional studies with larger national samples, researchers have found a positive relationship between food insecurity and intimate partner violence.[17, 18] Yet, there is an urgent need for studies that utilize longitudinal samples of women to confirm these cross-sectional findings and additionally, to examine the association between persistent exposure to food insecurity and women’s risk for violence. Persistent life conditions, such as chronic food insecurity, can be even more detrimental to health than acute crises or events [19]. Moreover, chronic food insecurity can adversely affect psychological, physical, and social well-being—including interpersonal relationships [20]. To date, cross-sectional studies have been unable to disentangle persistent from shorter-term food insecurity and its association with violence.

Even less is known about the role of HIV status in the association between food insecurity and violence against women. In general, HIV-positive women experience high rates of violence, poverty, and food insecurity [4], and are at risk for stigma, discrimination, and poor mental and physical health because of their HIV status [21, 22]. Food insecurity is associated with higher CD4 cell counts, poorer adherence to ART, decreased viral suppression, and increased morbidity and mortality [4]. According to the theory of syndemics [23], HIV infection may magnify the association between food insecurity on violence and lead to synergistic relationships between mental and physical health. Yet only one study has considered the role of HIV infection in its evaluation of food security and violence. Montgomery and colleagues examined correlates of violence, including food insecurity, using cross-sectional data from the Women’s HIV Seroincidence Study in the U.S. (N = 2,099) [18]. There were only 30 HIV-positive women in the sample making it difficult to examine the role of HIV status.

In the current study, we used longitudinal data from a national cohort study of women at risk or living with HIV to: (1) investigate the association of current and prior food insecurity (i.e., persistent food insecurity) with sexual, physical, and psychological violence; and (2) examine whether the association between food insecurity and violence differs by HIV status. For aim 1, we hypothesized that women who are food insecure would be at higher risk for violence as compared to women who are food secure. We also hypothesized a dose-response relationship would exist between food insecurity and violence in terms of the duration (i.e. persistence) and severity of food insecurity. For aim 2, we hypothesized that the association between food insecurity and violence would be stronger among women who are HIV-positive.

## Materials and methods

### Study population and procedures

Longitudinal data came from the U.S. Women’s Interagency HIV Study (WIHS), a multi-site, prospective cohort study of women at risk for or living with HIV. Study procedures and eligibility criteria have been described elsewhere [24]. WIHS participants are representative of the demographic profiles of women living with HIV in the U.S. and were recruited across 10 cities: Bronx, NY; Brooklyn, NY; Washington, D.C.; Chicago, IL; San Francisco, CA; Chapel Hill, NC; Miami, FL; Birmingham, AL; Jackson, MS; Atlanta, GA. Women completed interviewer-administered questionnaires every six months on demographic characteristics, mental health, violence, and other psychosocial factors, and had a brief clinical examination with laboratory tests. From April 2013 to April 2016, a module on food insecurity was added to the questionnaire. Refer to the WIHS website for survey instruments corresponding to visits 38–43 (https://statepi.jhsph.edu/wihs/wordpress/data-collection-forms). Food insecurity data were collected over six study visits every six months. Participants provided written informed consent and were compensated for participation. The protocol was approved by the WIHS executive committee and the following institutional review boards (IRB) at each site: the Human Research Protection Program at the University of California San Francisco, the Einstein IRB at the Albert Einstein College of Medicine, the SUNY Downstate Medical Center IRB & Privacy Board, the IRB at Rush University Medical Center, the Cook County Health & Hospitals IRB, the Georgetown University IRB, the Inova Health System IRB, the Maryland Department of Health IRB, the Sutter Health IRB, the Alameda Health Systems IRB, the University of North Carolina Biomedical IRB at Chapel Hill, the Emory IRB at Emory University, the University of Miami IRB, the University of Alabama at Birmingham IRB for Human Use, and the University of Mississippi Medical Center IRB.

### Measures

The primary explanatory variable was food insecurity, as measured by the U.S. Household Food Security Survey Module (HFSSM) [25]. The HFSSM consists of 18 items (e.g., “We worried whether our food would run out before we got money to buy more.”) that assessed food insecurity in the past six months. The HFSSM has been shown to accurately identify food-insecure households and predict known determinants and consequences [26]. We used the standard HFSSM scoring algorithm to categorize individuals as having high food security, marginal food security (some uncertainty about food supplies, but little to no indications of change in diet or food intake), low food security (reduced quality, variety, or desirability of diet, but little or no indication of reduced food intake) or very low food security (multiple indications of disrupted eating patterns and reduced food intake). Marginal, low, and very low food security represent increasing levels of food insecurity. In this study, the HFSSM scale demonstrated high reliability (alpha = 0.91).

The primary outcome variables were: (1) sexual or physical violence; and (2) psychological violence. Sexual violence was assessed with the question, “Since your (month) study visit, has anyone pressured or forced you to have sexual contact? By sexual contact, I mean them touching your sexual parts, you touching their sexual parts, or sexual intercourse.” Response options were: 1) yes, 2) no, 3) don’t know, and 4) declined to answer. Physical violence was assessed with the question, “Since your (month) study visit, have you experienced serious physical violence (physical harm by another person)? By that I mean were you ever hurt by a person using an object or were you ever slapped, hit, punched, kicked.” Due to the low number of events, we combined these two questions into a single binary variable. The two questions on sexual and physical violence were in reference to “any person,” which could include both partners and non-partners. The questions on psychological violence were in reference to a “current or previous” partner. This was assessed with seven items (yes/no) such as whether a partner “threatened to hurt you or kill you, “prevented you from leaving or entering the house,” and “prevented you from seeing your friends.” Women who responded yes to any of the seven items were coded as having experienced psychological violence.

### Analysis

Analysis models included all data collected over six visits. We used two-level logistic regression to model the association between food insecurity and experiences of violence, with women as a random effect. We used random rather than fixed effects because we expected that time-invariant differences between individuals could influence whether women experience violence, and we wanted to directly estimate the effects of these variables on women’s risk for violence. Random effects assume that the error term across individuals is not correlated with the predictors, thus allowing for time-invariant variables to play a role as explanatory variables.[27] We included a lagged variable for prior food insecurity in addition to a variable for current food insecurity. Since the models were additive, this allowed us to examine the association between persistent food insecurity (prior and current) and violence. The term “current” refers to the same visit at which violence is also assessed, whereas “prior” refers to a lagged visit which occurred six months before the current visit.

The models adjusted for potential confounders based on existing literature and theory: marital status (binary variable consisting of married/cohabitating or unmarried), currently in a relationship with a partner (yes/no), number of dependent children under care (continuous variable), employment status (employed/unemployed), annual household income (variable consisting of eight income categories), race/ethnicity (categorical variable with four types), stable housing status (yes/no), age (continuous variable), depressive symptoms (continuous variable measured using the Center for Epidemiological Studies Depression scale[28]), alcohol use (categorical variable with four levels: non-drinker, 0–7 drinks/week, 7–12 drinks/week, >12 drinks/week), any illicit drug use (such as cocaine, crack, heroin, amphetamines, club drugs, or methadone, but excluding marijuana), transactional sex (exchanging sex for food, money, or shelter; categorical variable with three levels: not having any sex; had sex, but no transactional sex; had transactional sex), and HIV status (positive/negative). Finally, to test for statistical interaction between HIV status and food security, we included an interaction term in the multivariable models.

Food insecurity information was available on 2,553 women at the baseline visit (Table 1). The final multivariable analysis used longitudinal data from 2,343 women who had complete data on all variables. Those excluded due to missing data (less than 10%) did not differ on key variables. All analyses were performed using Stata 15.

**Table 1:** Baseline characteristics of participants in the women's interagency HIV study (Food insecurity sub-study): United States, 2013–16.

	Full Baseline Sample	Food Insecure^a^	Food Secure^b^
Variable	No.	No. (%)	No. (%)
*All*	2553		
Age at visit (years)			
<50	1306	854 (52.49)	452 (48.81)
≥50	1247	773 (47.51)	474 (51.19)
Education level			
Less than high school	832	572 (35.20)	260 (28.08)
High school education	804	545 (33.54)	259 (27.97)
Some education beyond high school	915	508 (31.26)	407 (43.95)
Income per year, $			
≤ 12000	1311	659 (58.63)	652 (46.54)
> 12000	1214	465 (41.37)	749 (53.46)
Employed			
Yes	891	307 (27.07)	584 (41.18)
No	1661	827 (72.93)	834 (58.82)
Race/ethnicity			
Non-Hispanic White	255	157 (9.47)	101 (10.91)
Non-Hispanic Black	1829	1169 (71.85)	660 (71.27)
Hispanic	377	237 (14.57)	140 (15.12)
Other	92	67 (4.12)	25 (2.70)
Marital status			
Married	543	221 (19.59)	322 (22.90)
Unmarried but living with partner	240	94 (8.33)	146 (10.38)
Unmarried	1751	813 (72.07)	938 (66.71)
Currently partnered			
Yes	1577	669 (59.05)	908 (64.03)
No	974	464 (40.95)	510 (35.97)
Housing status			
Stable	2498	1097 (96.74)	1401 (98.80)
Unstable	54	37 (3.26)	17 (1.20)
Depressive symptoms^c^			
Yes	891	558 (49.25)	316 (22.28)
No	1660	575 (50.75)	1102 (77.72)
Alcohol use since last visit			
No alcohol use	1279	556 (49.03)	723 (50.95)
>0–7 drinks/week	884	391 (34.48)	493 (34.74)
>7–12 drinks/week	137	57 (5.03)	80 (5.64)
>12 drinks/week	253	130 (11.46)	123 (8.67)
Any illicit drug use (excluding marijuana)			
Yes	331	190 (16.75)	141 (9.94)
No	2222	944 (83.25)	1278 (90.06)
Exchanged sex for money, drugs, or shelter			
No sexual intercourse since last study visit	842	359 (31.66)	483 (34.04)
Had sex, but no transactional sex	1623	718 (63.32)	905 (63.78)
Transactional sex	88	57 (5.03)	31 (2.18)
HIV status			
HIV-positive	1803	1135 (69.76)	668 (72.14)
HIV-negative	750	492 (30.24)	258 (27.86)
Sexual or physical violence			
Yes	114	83 (7.33)	31 (2.19)
No	2437	1050 (92.67)	1387 (97.81)
Psychological violence			
Yes	112	77 (6.80)	35 (2.47)
No	2439	1056 (93.20)	1383 (97.53)

*Notes*: HIV = Human Immunodeficiency Virus

^a^ Food insecure women were defined as having marginal, low, or very low food security.

^b^ Food secure women were defined as having high food security.

^c^ Depressive symptoms was defined as having a score of ≥ 16 on the Center for Epidemiologic Studies of Depression scale.

## Results

At baseline (N = 2,553), the mean age was 47 years old, 64% had a high school education or less, 72% were non-Hispanic Black, and 62% reported having a current partner (Table 1). Consistent with the WIHS study design, 71% were HIV-positive. Almost 45% of women experienced food insecurity (defined as having marginal, low, or very low food security) in the past six months. In the past six months, 4.5% of women experienced sexual or physical violence and 4.4% of women experienced psychological violence.

The bivariate logistic regression models showed that women who were HIV-positive were less likely to experience sexual or physical violence (odds ratio (OR) = 0.60; 95% confidence interval (CI): 0.41, 0.88) and were less likely to experience psychological violence (OR = 0.55; 95% CI: 0.36, 0.83), compared to HIV-negative women. There was a significant dose-response relationship between current food security and both measures of violence (Table 2). The odds of experiencing sexual or physical violence were 2.18 times greater for women with marginal food security (95% CI: 1.47, 3.23), 2.87 times greater for women with low food security (95% CI: 1.96, 4.20), and 8.64 times greater for women with very low food security (95% CI: 6.04, 12.36), as compared to women with high food security. Similarly, a dose-response relationship was observed between current food security and psychological violence; corresponding unadjusted ORs for marginal, low, and very low food security were 2.84 (95% CI: 1.89, 4.26), 3.14 (95% CI: 2.08, 4.72), and 6.70 (95% CI: 4.48–10.02). There was also a significant dose-response relationship between prior food security and both measures of violence (see Table 2).

**Table 2:** Bivariate associations between food insecurity and violence: Women's interagency HIV study, United States, 2013–16.

Variable	Sexual or Physical Violence				Psychological Violence			
Food insecurity^a^	OR	95% CI	N	N reporting violence	OR	95% CI	N	N reporting violence
Current visit								
High food security	Ref	Ref	7406	124	Ref	Ref	7405	110
Marginal food security	2.18	1.47–3.23***	1535	55	2.84	1.89–4.26***	1535	60
Low food security	2.87	1.96–4.20***	1471	72	3.14	2.08–4.72***	1471	69
Very low security	8.64	6.04–12.36***	1230	137	6.70	4.48–10.02***	1229	107
Number of observations across study (N)			11642	388			11640	346
Number of women			2551				2551	
Prior (lagged) visit								
High food security	Ref		5744	88	Ref		5742	71
Marginal food security	2.17	1.34–3.50**	1081	33	2.02	1.20–3.41**	1082	36
Low food security	3.47	2.22–5.42***	1060	44	2.54	1.52–4.24***	1059	44
Very low security	7.20	4.69–11.08***	827	91	5.66	3.43–9.33***	826	70
Number of observations across study (N)			8712	256			8709	221
Number of women			2363				2363	

*Notes*: CI = Confidence Interval; HIV = Human Immunodeficiency Virus; N = Number of observations; OR = Odds Ratio

*P<0.05

**P < .01

***P < .001.

^a^Four separate models were fit for each combination of violence and current/prior food insecurity. Crude odds ratios computed using N and the number reporting violence will differ slightly from the odds ratios reported because the multi-level logistic regression models control for the clustering of visits (level 1) within women (level 2).

After adjusting for potential confounders, the odds of experiencing sexual or physical violence were 3.12 times greater for women with very low food security at the current visit as compared to women with high food security at the current visit (95% CI: 1.88, 5.19; see Table 3). The odds of experiencing sexual or physical violence were 7.05 times greater for women with very low food security at both the current and prior visits compared to women with high food security at both visits (the natural logarithms of 3.12 and 2.26 for current and prior very low food security were summed and then exponentiated to obtain 7.05). Similarly, the odds of experiencing psychological violence were 3.03 times greater for women with very low food security at the current visit compared to women with high food security at the current visit (95% CI: 1.67, 5.50). The odds of experiencing psychological violence were 5.72 times greater for women with very low food security at both visits compared to women with high food security at both visits. Other covariates were significantly associated with both forms of violence: age, employment, and stable housing were negatively associated with violence, while having a current partner, depression, alcohol use, engaging in transactional sex, and any illicit drug use were positively associated with violence (see Table 3). HIV seropositive status was no longer significantly associated with violence in the adjusted models (the association became non-significant when adding prior food insecurity to the models containing all other variables including income). There was no evidence of statistical interaction between HIV status and food insecurity on violence.

**Table 3:** Adjusted Odds Ratios (AOR) and 95% Confidence Intervals (CI) for associations between food insecurity and violence: Women's interagency HIV Study, United States, 2013–16.

Variable	Sexual or Physical Violence		Psychological Violence	
**Food insecurity**	AOR	95% CI	AOR	95% CI
Current visit				
High food security	Ref	Ref	Ref	Ref
Marginal food security	1.37	0.82–2.32	2.12	1.22–3.69**
Low food security	1.29	0.78–2.14	1.72	0.98–3.00
Very low food security	3.12	1.88–5.19***	3.03	1.67–5.50***
Prior (lagged) visit				
High food security	Ref	Ref	Ref	Ref
Marginal food security	1.65	1.05–2.93	1.69	0.97–2.94
Low food security	1.58	1.04–2.77	1.06	0.60–1.87
Very low food security	2.26	1.49–4.02***	1.89	1.07–3.33*
**Socio-demographics**				
Married/cohabitating (yes/no)	0.88	0.56–1.37	1.11	0.67–1.82
Has a current partner (yes/no)	1.91	1.28–2.86**	2.17	1.38–3.43***
Number of children under care	1.16	0.98–1.38	1.19	0.99–1.43
Age at visit	0.96	0.94–0.99	0.95	0.93–0.98***
Employed	0.43	0.26–0.72**	0.40	0.22–0.71**
Income	1.05	0.95–1.18	1.05	0.93–1.19
Stable housing (yes/no)	5.31	2.44–11.58***	2.11	0.75–5.90
Race/ethnicity				
Non-Hispanic White	Ref	Ref	Ref	Ref
Non-Hispanic Black	0.91	0.47–1.75	0.51	0.24–1.06
Hispanic	0.44	0.17–1.10	0.42	0.16–1.13
Other	2.48	0.92–6.67	2.85	0.96–8.51
**Other covariates**				
Depression (yes/no)	1.05	1.04–1.07***	1.07	1.05–1.08***
Alcohol use				
No alcohol use	Ref	Ref	Ref	Ref
>0–7 drinks/week	1.60	1.06–2.40*	2.19	1.36–3.53***
>7–12 drinks/week	2.74	1.42–5.27**	2.58	1.19–5.60*
>12 drinks/week	2.06	1.19–3.57**	2.44	1.28–4.66**
Any illicit drug use (yes/no)	2.74	1.71–4.39***	2.29	1.31–3.99**
Transactional sex				
No sexual intercourse	Ref	Ref	Ref	Ref
Sex, but no transactional sex	3.21	1.97–5.25***	5.76	3.12–10.64***
Transactional sex	5.06	2.10–12.17***	5.12	1.75–14.95**
HIV positivity	0.92	0.60–1.39	0.87	0.53–1.42
		N		N
Number of women		2343		2343
Number of observations across study visits		8528		8526

*Notes*: AOR = Adjusted Odds Ratios; CI = Confidence Interval; HIV = Human Immunodeficiency Virus.

*P < .05

**P < .01

***P < .001

## Discussion

In this longitudinal cohort of women, many of whom were living with HIV, we found that food insecurity—either at the prior or current visit—was positively associated with current violence even after adjusting for socio-economic status. This finding held for sexual or physical violence, and psychological violence. We also provide the first longitudinal evidence that being food insecure at a prior study visit is associated with subsequent experiences of violence. Additionally, women who experienced persistent, severe food insecurity were more likely to experience violence than women who experienced shorter periods of severe food insecurity.

There are several explanations for our findings. First, although food insecurity differs from poverty and we controlled for markers of poverty in the analysis, parallels can be drawn from existing literature on violence and poverty of which more is known. Poverty places women at increased risk for sexual and physical violence through the path of stress [8]. Food insecurity could also invoke stress by causing hunger and worry about having sufficient access to food, and feelings of deprivation and alienation [1], these in turn may serve as a trigger for violence in families or couples [9, 10]. Second, food insecurity may make it difficult for women to leave abusive relationships due to dependence on their partners for food [29]. Finally, food insecurity may be related to violence through sexual behaviors such as transactional sex. Transactional sex has been described as a survival means to obtain food [29] and women who are food insecure are more likely to engage in risky sexual behaviors including transactional sex [30, 31]. Other research has found a positive association between transactional sex and multiple forms of intimate partner violence [32, 33]. Not only can food insecurity, transactional sex, and other psychosocial factors such as substance abuse independently contribute to women’s risk for violence, but can co-occur together as a syndemic and have a compounding effect [34]. Finally, we found that persistent food insecurity is a stronger predictor of violence than shorter-term food insecurity. Persistent food insecurity that is difficult to overcome may cause significant stress, feelings of powerlessness, and social isolation—conditions that may ultimately foster violence [20].

This is also the first study to examine the role of HIV status in the association between food insecurity and violence. Contrary to our hypothesis, HIV status did not amplify the association between food insecurity and violence. HIV-positive women reported significantly less alcohol use, drug use, and risky sex, and may have better access to social services (including counseling for victims of violence) due to their HIV status and engagement in HIV care. This may be especially relevant to HIV-positive women in WIHS, many of whom have been receiving services for HIV infection for years. Use of services for substance abuse and mental health among HIV-positive individuals is high and may be higher in comparison to the general population [35]. It is possible that for some HIV-positive women, these services may provide skills and resources needed to avoid abusive relationships.

### Public health implications

Given this evidence showing a strong, positive association between food insecurity and violence, and the importance of persistent food insecurity, structural interventions to improve access to and availability of food may also prevent violence. While economic empowerment interventions (e.g., microfinance) have been developed to address women’s risk for violence, these interventions do not specifically target food insecurity. Targeted food assistance (e.g., food banks, meal delivery programs), supplemental nutritional assistance programs, and livelihood or vocational training programs are promising options to address food insecurity [36] and have been recommended for other vulnerable populations living with HIV such as drug users [37, 38]. For people living with HIV in other countries, efforts are underway to provide integrated, nutritional support within HIV clinical care [39] and similar models could be adapted for U.S. women. There may be a need for multi-level programs that merge structural-level interventions with interpersonal interventions to improve couple communication around financial stress and food acquisition. Couples-based interventions have been shown to improve communication and problem-solving, and reduce sexual risk behaviors, and could be adapted [40]. Because recurring exposure to food insecurity had a stronger association with violence, interventions should strive for early prevention of food insecurity. Screening for food insecurity during clinic visits could be an important opportunity to identify food insecurity if appropriate interventions were available (e.g., referrals to food programs)—and to prevent long-term struggles with food insecurity. Evidence from this study also indicates that efforts should focus on impoverished women in general regardless of HIV status.

### Limitations

Our measures of physical and sexual violence did not allow us to assess who perpetrated violence against women. However, the most common form of violence against women worldwide is intimate partner violence, which may have comprised a large proportion of the violence captured in our measure. Future studies should confirm our findings using questions specifically asking about intimate partner violence. The prevalence of violence in this sample was also low in comparison to other studies [18]. This could be attributed to social desirability bias and the older age of the cohort. Other studies have found that the risk for violence is highest among young women [41]. Moreover, an earlier study using WIHS data (where participants were 20 years younger) found much higher rates of violence, with 21–28% of women having experienced sexual or physical violence in the past year [42]. It is also possible that there is self-selection bias due to having participated in WIHS over many decades, which could be difficult for women experiencing violence. Finally, the majority of the sample were from northern sites, where rates of violence are generally lower than in the southern sites. Consistent with the HIV epidemic among women in the U.S., the WIHS sample was also predominantly African American (72%) and the findings may be most relevant to this racial/ethnic group.

### Conclusions

We found a strong, positive relationship between food insecurity and violence in a longitudinal cohort of women with and at risk for HIV in the U.S. Cumulative exposure to food insecurity was a stronger predictor of violence than at a single time point. HIV-positive status did not play a role in these associations. Efforts to address food insecurity must consider the interpersonal harms that U.S. women with food insecurity experience, such as violence. Programming to address chronic exposure to food insecurity may yield substantial reductions in women’s experiences of violence and should be investigated in future research.
